# Exploring the Impact of Glycemic Control on Diabetic Retinopathy: Emerging Models and Prognostic Implications

**DOI:** 10.3390/jcm13030831

**Published:** 2024-01-31

**Authors:** Nicola Tecce, Gilda Cennamo, Michele Rinaldi, Ciro Costagliola, Annamaria Colao

**Affiliations:** 1 Unit of Endocrinology, Dipartimento di Medicina Clinica e Chirurgia, Federico II University Medical School of Naples, 80131 Napoli, Italy; teccenicola@gmail.com (N.T.); colao@unina.it (A.C.); 2 Department of Neurosciences, Reproductive Sciences and Dentistry, University of Naples “Federico II”, 80131 Naples, Italy; ciro.costagliola@unina.it; 3 Department of Neurosciences, Reproductive and Odontostomatological Sciences, Federico II University, 80131 Naples, Italy

**Keywords:** diabetes (T1D), epidemiology, microangiopathic complications, DR, non-proliferative DR (NPDR), proliferative DR (PDR), advanced hybrid closed-loop (AHCL) systems, continuous glucose monitoring (CGM), personalized medicine, machine learning, open-source HCLSs, AndroidAps, neovascularization, diabetes management

## Abstract

This review addresses the complexities of type 1 diabetes (T1D) and its associated complications, with a particular focus on diabetic retinopathy (DR). This review outlines the progression from non-proliferative to proliferative diabetic retinopathy and diabetic macular edema, highlighting the role of dysglycemia in the pathogenesis of these conditions. A significant portion of this review is devoted to technological advances in diabetes management, particularly the use of hybrid closed-loop systems (HCLSs) and to the potential of open-source HCLSs, which could be easily adapted to different patients’ needs using big data analytics and machine learning. Personalized HCLS algorithms that integrate factors such as patient lifestyle, dietary habits, and hormonal variations are highlighted as critical to reducing the incidence of diabetes-related complications and improving patient outcomes.

## 1. Introduction

### 1.1. Brief Overview of Type 1 Diabetes (T1D)

Type 1 diabetes (T1D) is a chronic autoimmune condition wherein the body’s immune system erroneously attacks and destroys insulin-producing beta cells in the pancreas. The exact etiology of this autoimmune reaction is not fully understood but is believed to involve a combination of genetic predisposition and environmental triggers. The resultant insulin deficiency leads to elevated blood glucose levels, necessitating lifelong insulin therapy [1]. T1D is a global health concern, affecting individuals across all ages and ethnicities, although its prevalence varies geographically [2,3]. In recent decades, the incidence of T1D has been increasing worldwide, posing challenges not only to healthcare systems but also highlighting the influence of environmental and lifestyle factors [4]. The management of T1D and its complications has significant economic implications, particularly in resource-limited settings, where access to essential medications and monitoring technologies may be constrained [5,6,7]. Over the past two decades, the prevalence of T1D has steadily increased, with an estimated 8.75 million people with T1D in 2022 [4]; of these, there were 530,000 incident cases of T1D in 2022; 35,000, on the other hand, is the estimated number of patients under 25 who died due to missed diagnosis of T1D [4]. This is of particular concern because patients with T1D have a life expectancy on average 11 years shorter than non-diabetic patients, mainly due to the macroangiopathic complications of T1D [8,9]. Living with T1D requires diligent and continuous management, which can be burdensome, especially for young patients. The need for regular blood glucose monitoring, insulin administration, dietary management, and physical activity requires significant lifestyle adjustments. Additionally, the psychological impact of managing a chronic condition can include stress, anxiety, and depression, underscoring the need for comprehensive care that includes mental health support [10].

### 1.2. Importance of Understanding T1D and Its Microangiopathic Complications

Although the use of intensive insulin therapy since the DCCT trial has greatly reduced the incidence of micro- and macroangiopathic complications of diabetes [11], in these patients, a reduced life expectancy and an increased prevalence of micro- and macroangiopathic complications remain even when glycemic compensation (estimated with Hba1c) appears optimal [8,12,13]. Chronic glycemic alteration, in addition to the frequent long disease duration in patients with T1D, can also lead to microangiopathic complications, including neuropathy, nephropathy, and diabetic retinopathy (DR) [14]. DR is a leading cause of blindness in adults, and diabetic nephropathy is a leading cause of end-stage renal disease [15,16]. In 2004, the direct costs associated with DR were estimated to be USD 493 million per year, although it should be noted that this figure was before the introduction of antiangiogenic drugs, which also have a significant impact on the cost of this disease [17]. In the meantime, the prevalence of macular edema is projected to increase by 25 percent over the next 10 years, placing an even greater economic burden on international systems [18]. In this context, it is essential to better understand the etiopathogenesis of this disease to improve its prevention. The high prevalence of DR and diabetic nephropathy in patients with T1D cannot be explained solely by the role of hyperglycemia: instead, multiple studies point to a multifactorial etiopathogenesis, which frequently leads to the association between retinopathy and diabetic nephropathy, so much so that the term “renal-retinal syndrome” [19,20] was coined, in which microvascular endothelial dysfunction is a common pathogenetic factor in the early development of the two diseases: the endothelial cells of the microcirculation are the main target of hyperglycemia-related damage through a series of pathways including the accumulation of advanced glycation end products (AGEs) and the increase of receptors for them, activation of the polyol pathway, and stimulation of PKC subtypes and the aminohexose signal pathway [19,20]. This review will mainly focus on the role of glycemic alterations in the etiopathogenesis of DR and how new models of glycemic management may play a role in the progression and prognosis of this disease.

### 1.3. Advancements in Treatment, Research Gaps, and Future Directions

Remarkable technological advancements have transformed T1D management. Continuous glucose monitoring (CGM) systems and advanced insulin delivery methods, such as insulin pumps and hybrid closed-loop systems, are changing the landscape of diabetes care [21]. These technologies not only improve glycemic control but also enhance the quality of life by offering greater flexibility and reducing the burden of constant monitoring and adjustments [21].

Despite these advancements, significant gaps remain in our understanding of T1D, particularly in its early detection, prevention, and the full integration of technological solutions in diabetes care. Future research is poised to explore the genetic and environmental factors contributing to T1D, develop more refined treatment protocols, and fully harness the potential of artificial intelligence and machine learning in diabetes management.

## 2. Diabetic Retinopathy: Clinical Features and Stages

### 2.1. Non-Proliferative Diabetic Retinopathy (NPDR) and Proliferative Diabetic Retinopathy (PDR)

**Figure 1:**
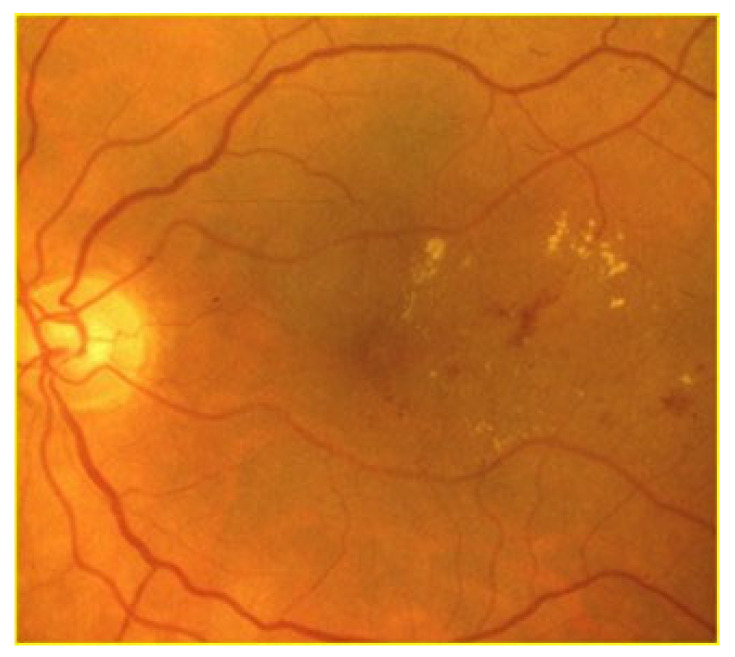
Non-proliferative diabetic retinopathy with retinal hemorrhages and hard exudates.

**Figure 2:**
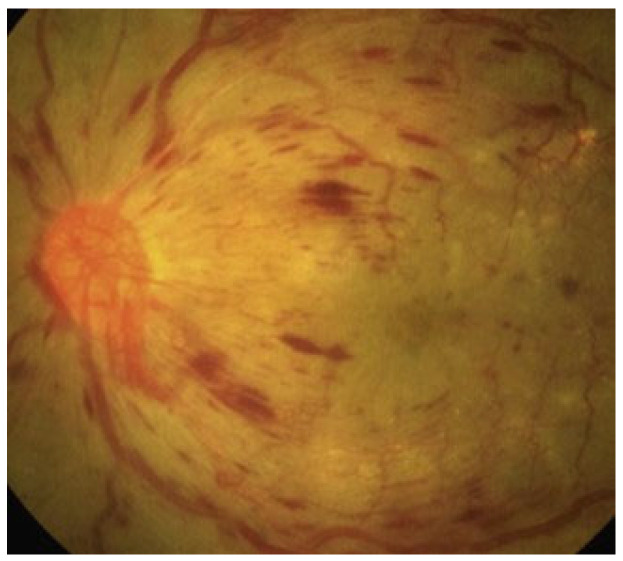
Proliferative diabetic retinopathy with retinal hemorrhages and retinal and papillary new vessels.

Clinically, DR is categorized into two distinct stages. The initial stage, known as non-proliferative DR (NPDR), is characterized by increased vascular permeability and the occlusion of retinal capillaries (Figure 1). During funduscopic examinations in this stage, microaneurysms, hemorrhages, and hard exudates may be observed, though patients typically do not experience symptoms at this point [22]. Progressing to the more advanced stage, proliferative DR (PDR) is marked by a critical phenomenon: neovascularization (Figure 2). This stage is a significant escalation in severity, as the growth of abnormal new blood vessels can lead to severe visual impairment. These vessels, forming in the vitreous or on the retina, are prone to causing complications such as vitreous hemorrhage or even retinal detachment, posing a serious threat to vision [23].

### 2.2. Diabetic Macular Edema (DME)

**Figure 3:**
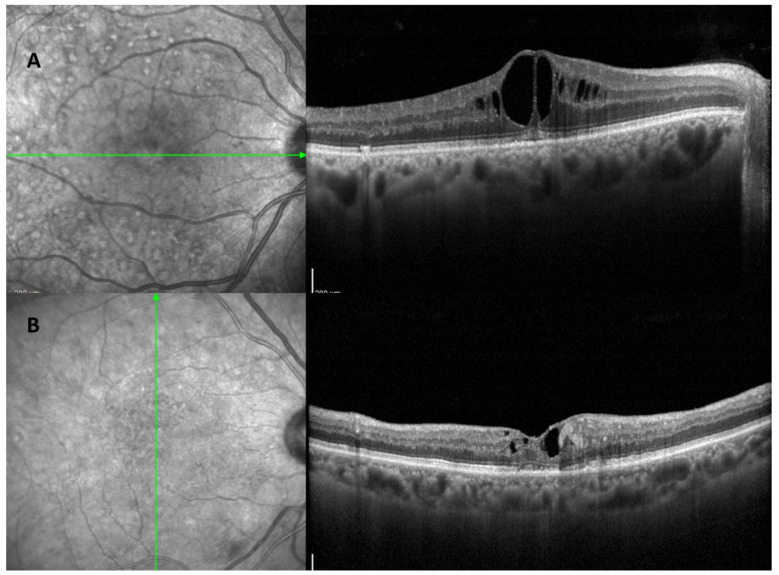
(**A**): OCT scan pass through the fovea shows an inflammatory diabetic macular edema (DME) phenotype characterized by hyper-reflective spots and intraretinal cysts with increases in central subfoveal thickness (**B**): OCT scan through to the fovea reveals a vascular DME phenotype characterized by small cystoid space and hard exudates.

Diabetic macular edema (DME) is the primary cause of vision loss in diabetic retinopathy (DR) [24]. It involves the macula swelling and thickening due to fluid buildup, which is linked to blood–retinal barrier (BRB) dysfunction [24] (Figure 3). The Early Treatment Diabetic Retinopathy Study (ETDRS) describes DME as either retinal thickening or the presence of hard exudates near the center of the macula [25]. DME can develop at any DR stage, leading to decreased visual acuity or visual distortion. It is especially prevalent in working-age people with type 2 diabetes. Globally, DME affects about 6.8% of individuals [2], with its prevalence increasing to about 30% in those who have had diabetes for over 20 years, and up to 71% in those with proliferative DR [26].

### 2.3. Role of Dysglycemia in the Pathogenesis of Diabetic Retinopathy

The pathogenesis of DR is complex and multifactorial, with the hyperglycemic state associated with diabetes playing a pivotal role in inducing microangiopathy. Hyperglycemia activates different metabolic pathways leading to increased hypoxia, reactive oxygen species formation, and inflammation [27,28,29]. Hyperglycemia leads to altered retinal vascular flow and dilation, probably adapted to compensate for the metabolic deficit in the diabetic patient [30]. At the same time, hyperglycemia is responsible for pericyte apoptosis, both in vitro and in vivo [31]. The lack of structural support to the capillaries provided by pericytes results in the process of aneurysmal dilatation of the capillaries, one of the first clinical signs of DR [32]. At the same time, hyperglycemia is associated with endothelial cell apoptosis and basement membrane thickening, leading to BRB dysfunction and subsequent retinal ischemia secondary to capillary occlusion [33]. Ischemia is followed by upregulation of VEGF through activation of hypoxia-inducible factor 1 [34]. VEGF promotes increased vascular permeability and endothelial cell proliferation, and its elevated levels have been documented in the retina of diabetic rats and in diabetic patients with DME and PDR [35,36]. In vitro studies have shown that hyperglycemia also plays a role in the induction of mitochondrial dysfunction leading to apoptosis of retinal neurons [37]. Hypoglycemia and glycemic variability are also thought to play a role in promoting the development of DR [38,39,40], with increasing support for the idea that it is not so much necessary to only lower blood glucose levels as to bring them as close as possible to those of an euglycemic patient but to increase the general time spent in euglycemia by the patient, taking also into account the time spent in hypoglicemia and the glycemic variance [39,40]. The use of artificial pancreas systems has been shown to reduce the prevalence and severity of hypoglycemia and glycemic variance [41,42,43] and is a potential tool for improving overall prognosis in diabetes and specifically in DR.

### 2.4. Role of Inflammation and Retinal Degeneration in the Pathogenesis of Diabetic Retinopathy

Inflammation significantly contributes to the development and continuation of DR [29]. It occurs when retinal cells, like pigment epithelium cells, Müller cells, and activated microglia, release inflammatory substances such as cytokines, chemokines, and growth factors. These substances disrupt the connections between endothelial cells, cause the loss of pericytes (cells that support capillaries), and lead to leukostasis (a slowing or stopping of blood flow), all of which affect the blood–retinal barrier and increase vascular permeability. This process is driven by an elevated production of the vascular endothelial growth factor (VEGF), culminating in the development of DME [27,28,44]. Another key factor in the progression of DR is retinal neurodegeneration, which occurs from the earliest stages of the disease [45]. As early as one month after induction of diabetes in streptozotocin-treated rats, apoptosis of retinal neurons can be observed [45], and increased levels of pro-apoptotic molecules and increased production of reactive oxygen species (ROS), which are involved in retinal neuronal apoptosis, have been demonstrated [46]. Although retinal neurodegeneration is associated with DR, recent evidence suggests that in diabetic patients it precedes the typical changes observed at the ocular fundus in the retinal microcirculation [47] and, thus, represents a potential therapeutic target for early management of the disease.

## 3. Advancements in Diagnostic Techniques of Diabetic Retinopathy

Recent advances in retinal imaging have greatly improved the early detection and management of diabetic retinopathy (DR). Color fundus photography, which is useful for screening and monitoring DR, shows key disease indicators such as microaneurysms, retinal hemorrhages, and changes in intraretinal capillaries and veins [48]. Fluorescein angiography (FA), although invasive and time-consuming, is effective in detecting capillary occlusion, new vessel growth, macular ischemia, and distinguishing between localized and widespread retinal edema [49]. This method distinguishes between focal macular edema, characterized by localized retinal thickening, and diffuse macular edema, characterized by generalized leakage and accumulation of dye in the retina. However, due to the invasive nature of FA, many ophthalmologists now rely solely on optical coherence tomography (OCT) for the management of DME [50]. OCT, which provides faster and more accurate assessments, measures central retinal thickness (CRT) to evaluate DME severity and treatment response. It also identifies different patterns of DME, such as diffuse retinal thickening, cystoid macular edema, and serous retinal detachment [51]. Recently, OCT has distinguished two types of DME based on unique biomarkers and treatment response: the vascular DME phenotype (Figure 3A), which is mainly due to inner blood–retinal barrier dysfunction and responds to anti-VEGF therapy, and the inflammatory DME phenotype (Figure 3B), which typically responds better to steroids [50,52,53]. The latter is linked to elevated cytokine levels in eye fluids, contributing to retinal changes [50]. Features like multiple hyper-reflective foci and serous retinal detachment, observed in 36.5% of DME eyes, are considered inflammation indicators, associated with increased hyper-reflective foci and interleukin-6 levels [50,54].

## 4. Advanced Hybrid Closed-Loop (AHCL) Systems and T1D

### 4.1. Intensive Insulin Therapy and Use of Continuous Glucose Monitors

In recent years, the care of patients with T1D has undergone a remarkable revolution. Intensive use of exogenous insulin has been shown to slow the progression of hyperglycemia-related complications in patients with T1D, improving quality of life and reducing associated costs; however, it increases the risk of hypoglycemia [55]. Thanks to major advances in blood glucose monitoring and insulin delivery technologies, a remarkable leap forward has been made in the management of diabetes. These breakthroughs have paved the way for a significant transformation in the management of DMT1, offering patients new opportunities to achieve glycemic control goals and improve their overall quality of life. The introduction of CGM systems has shown that glycate alone is not sufficient to assess the adequacy of glycemic compensation, but great importance should also be given to time spent in hypoglycemia (<70 and <54), time spent in hyperglycemia (<180 and >300), and glycemic variability: the use of these systems allows us not only to improve our ability to monitor diabetes, but also to improve the compensation of diabetes itself [56].

### 4.2. Continuous Subcutaneous Insulin Infusion (CSII)

The use of continuous subcutaneous insulin delivery (CSII) devices has revolutionized insulin therapy in recent years; compared to multiple injection therapy (MDI), in which the patient takes three prandial ultra-rapid insulins and a daily basal insulin of approximately 24 hours’ duration [57], the pump allows the exclusive delivery of ultra-rapid insulin at rates programmed by the diabetologist [58], which vary according to the different circadian needs of patients, in addition to the prandial boluses that are routinely administered before meals by the CSII [58]. In these systems, it is often possible to schedule different daily insulin infusion profiles according to the different needs of the patient [58] (days when the patient works vs. weekends, days when menstruation occurs, days when the patient is sick). In addition, the CSII allows prandial insulin boluses to be delivered at different rates depending on the macronutrient composition of the meal and based on different rates of nutrient absorption by patients (e.g., due to gastropathy associated with autonomic neuropathy) [58]. A major advance in prandial bolus management has been the introduction of pump-integrated bolus calculators [58], which allow accurate calculation of the recommended bolus for the patient by taking into account the insulin still in circulation delivered by the CSII, the patient’s current measured blood glucose, target blood glucose, and patient-specific factors (periodically modified by the diabetologist) such as insulin sensitivity, insulin-to-carbohydrate ratio, and duration of insulin use [58]. Commercially available pumps can deliver insulin subcutaneously through a cannula attached to a catheter connected to the CSII reservoir, or they can be catheterless, with the reservoir connected directly to the subcutis through a needle and monitored by an external PDA or cell phone [58]. A major risk of using insulin pumps is the occurrence of diabetic ketoacidosis (DKA) due to malfunctioning of the pump, which can be limited by the use of continuous glycemic monitoring sensors, which have the capacity to alert the patient of CSII malfunctions through continuous detection of hypo/hyperglycemia in addition to the progressive improvements in patient education [59]. Supported by international guidelines, pump therapies have emerged as a highly recommended approach for people with DMT [58].

### 4.3. HCLSs and Glycemic Variability

**Figure 4:**
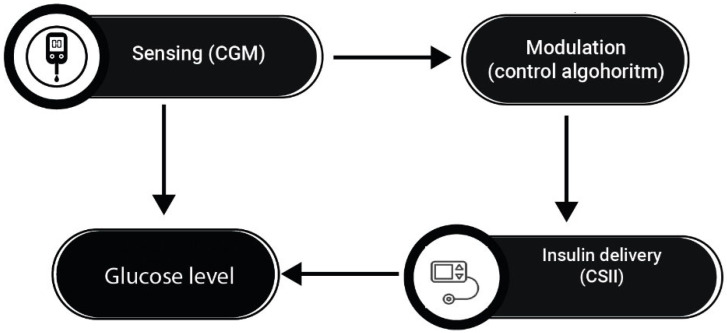
The figure shows how hybrid closed-loop pancreas systems use information on glucose levels to regulate insulin delivery.

Recently, hybrid closed-loop systems (HCLSs), also known as hybrid artificial pancreas or closed-loop glucose control systems, have been developed to safely and automatically control blood glucose levels by integrating a subcutaneous insulin infusion pump, a subcutaneous continuous glucose monitor (CGM), and a control algorithm that continuously calculates the amount of insulin to infuse into the patient (Figure 4) [58]. Use of the subcutaneous route for both glucose sensing and insulin delivery is preferred over the intravenous (IV) and intraperitoneal (IP) routes because of the convenience and safety of outpatient use [60]. The IV route can lead to frequent catheter problems such as intravenous migration and fibrin clot occlusion, while the IP route requires implantation and may increase the production of anti-insulin antibodies [60]. Based on CGM measurements and the microdoses that the pump can handle, an HCLS is able to deliver the amount of insulin that the patient needs at any given time. In addition to insulin, glucagon and amylin analog hormones have been considered to improve the performance and safety of HCLSs [61]. These systems have revolutionized glycemic control by automating insulin delivery in response to continuous glucose monitoring data. However, the complexity and heterogeneity of diabetes require a more personalized approach to further improve metabolic control and prevent dysglycemia. Currently, no HCLS considers the exact macronutrient composition of meals, only their carbohydrate content, which limits the effectiveness of the bolus predictor. Another important limitation of the algorithms that run these systems is that they do not take into account a number of anamnestic, anthropometric, and laboratory parameters to improve the pump’s ability to match insulin delivery to the patient’s blood glucose. No HCLS considers specific patient characteristics that may modify insulin sensitivity, such as the presence of dual diabetes, the sex of the patient, the presence of menopause or hypogonadism, or the presence of renal failure and/or liver cirrhosis. All of these factors, which are already measured to monitor the status of the diabetic patient and which affect the patient’s insulin sensitivity in different ways, are currently ignored by all commercially available HCLSs.

## 5. Technological Advancements in Diabetes Management: A Gateway to Preventing Diabetic Retinopathy and Other Microangiopatic Complications of T1D

### 5.1. Benefits of Using Continuous Glucose Monitors on Diabetic Retinopathy

The use of CGM systems and insulin pumps in the management of T1D has been shown to significantly improve glycemic outcomes and reduce the risk of microangiopathic complications such as diabetic retinopathy. A study that followed patients with T1D for 7 years found that those who initiated a CGM within the first year of diagnosis and continued to use it compared to patients who did not use CGM sensors showed a significantly better HbaA1c level [62]. This improvement was maintained throughout the 7-year follow-up period, suggesting a long-term benefit of CGM in glycemic control [62]. An association between worse glycemic control and DR was suggested from a study on 3262 T2D patients conducted in 2018: from this study emerged how the prevalence of DR decreased with higher TIR, and the severity of DR was inversely correlated with lower TIR [63]. In a 2023 retrospective study by Shah et al., 163 patients with T1D were studied, with CGM data collected for up to 7 years prior to eye examination: TIR, TITR, TAR, and mean glucose were associated with increased risk of incident DR in adults with T1D [64]. In another interesting study from 2013, 68 patients (35 with type 1 diabetes and 33 with type 2 diabetes) were monitored for six weeks with CGM sensors: from the study, emerged an association between glucose variability and RD, regardless of HbA1c [65].

TIR was even proposed as an useful marker for evaluating retinal functional changes in diabetic retinopathy patients: in a cross section study from 2023, including 100 eyes of non-DR patients and 60 eyes of DR patients, TIR showed a correlation with retinal mean sensitivity reduction in DR patients, suggesting a useful option for evaluating DR progression [66].

In conclusion, the integration of continuous glucose monitoring systems in the management of Type 1 diabetes not only enhances glycemic control but also plays a crucial role in mitigating the risk of diabetic retinopathy and other microangiopathic complications.

### 5.2. Benefits of Using HCLSs and Insulin Pumps on Diabetic Retinopathy

Studies have shown that the use of hybrid closed-loop (HCL) systems in people with type 1 diabetes (T1D) can significantly improve blood glucose control. A study focused on the real-world use of HCL systems in the U.S. showed that the overall glycemic control achieved with these systems was consistent with the results of pivotal trials [67]. This finding demonstrates the effectiveness of HCL systems in managing blood glucose levels in a real-world setting, consistent with the controlled conditions of clinical trials [67].

In addition, a comparative study highlighted that different technology modalities in T1D, including HCL systems, are associated with improved glycemic control [68]. This study suggests that HCL systems offer significant advantages in terms of time in range and hypoglycemia protection compared to other diabetes management technologies [68].

A 2023 study from Guo et al. of CSII systems explored the effects of different insulin therapies in children with T1DM on diabetic retinopathy [69]: from the study where 42 participants were treated with multiple daily injections (MDI) of insulin and 22 with CSII, it emerged that patients treated with insulin pumps had a better vessel density than patients treated with MDI, suggesting that CSII may be a better choice for T1DM children to prevent retinal complication [69].

In summary, the integration of CGM and insulin pump therapy in the management of T1D not only improves glycemic control, but also has the potential to significantly reduce the risk of developing microangiopathic complications such as diabetic retinopathy. This underscores the importance of early and sustained use of these technologies in the management of T1D.

## 6. Emerging Research and Future Perspectives

### 6.1. Open-Source HCLSs

**Figure 5:**
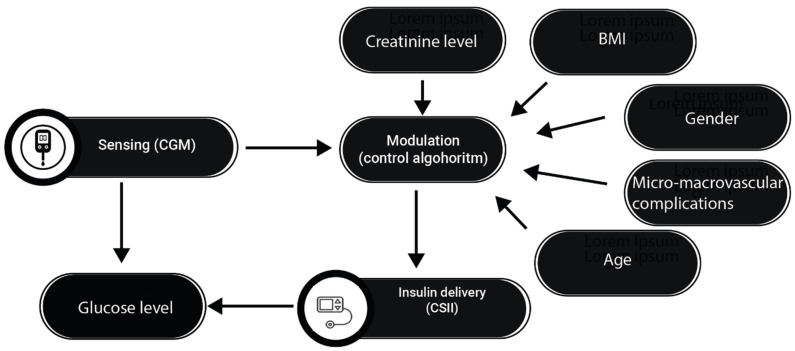
The figure shows how some possible factors could influence the algorithms that regulate blood glucose—these will be studied retrospectively in our study.

When we talk about open-source HCLSs, we are essentially referring to systems where the brain that controls the interaction between the CGM and CSII is an Android application, AndroidAps [70]. In these systems, whose primary goal is to reduce the cost of HCLSs and enable interoperability between the CGM and CSII, the system algorithm is open and potentially modifiable by a team of experienced researchers [70]. Although open-source artificial pancreases may appear to be equivalent in operation to traditional advanced HCLSs, the potential of the latter becomes apparent when considering the possibilities offered by modern big data analytics. In fact, with these specific pumps, it is not only possible to perform retrospective analyses to see how a series of parameters known to affect blood glucose levels can modify the circadian glycemic patterns of patients, but it is also possible to hypothesize that not only the classical parameters underlying pump operation (basal insulin, insulin sensitivity, insulin–carbohydrate ratio, insulin duration) could be modified by the diabetologist: the entire algorithm of the HCLS could potentially be modified to tailor it to each specific patient (Figure 5).

### 6.2. Prospects for Future Research and Development

It is, then, possible to hypothesize that the pattern of insulin release in the dialyzed patient may be modified on days when he or she is further from dialysis, and that the system’s response may be differentiated considering the patient’s lean and fat mass, age, hormonal profile, and comorbidities (e.g., in patients undergoing chemotherapy on days when they are taking cortisone). The innovation that these systems offer us is the possibility of directing clinical research towards using the complex interplay between patient history, anthropometric parameters, and instrumental studies in diabetes management in a structured way, to build algorithms that not only respond to changes in blood glucose levels to correct insulin delivery, but are also able, in the manner of a human diabetologist, to take into account all of the patient’s comorbidities to stitch together a specific therapy that takes into account all of the patient’s specific characteristics (Figure 5).

## 7. Limitations and Future Directions

Despite significant advances in the understanding and management of type 1 diabetes (T1D), there remain significant areas for improvement and research. A major limitation in current T1D management is the lack of a fully automated, adaptive insulin delivery system. While hybrid closed-loop systems represent a significant advance, they still require user intervention for mealtime insulin dosing and do not fully account for the dynamic and individualized nature of glucose metabolism. Future research should focus on developing more sophisticated algorithms that integrate a wider range of physiological parameters, such as stress levels, physical activity, and hormonal fluctuations, for a truly personalized and automated insulin delivery system. Another limitation lies in the scope of current research, which focuses predominantly on the physiological aspects of T1D: the psychosocial impact of T1D, particularly in children and adolescents, is also an area that needs more attention. Future interventions should emphasize not only the physical management of T1D but also the psychosocial aspects to ensure a holistic approach to patient care and should include psychological support and strategies to improve adherence and quality of life. In addition, while the review discusses advances in diagnostic techniques for diabetic retinopathy, it does not fully explore the limitations of these techniques in terms of availability, cost, and applicability at different stages of the disease. Further studies are needed to improve early detection methods and make them more accessible to diverse populations. Furthermore, the integration of big data and artificial intelligence into T1D management is promising but still in its early stages. The potential for these technologies to predict and prevent complications, personalize treatment plans, and improve overall disease management is immense and warrants further exploration. Finally, addressing disparities in T1D care is critical. Access to advanced treatment options and technologies is often limited by socioeconomic factors. Research and policy efforts should aim to reduce these disparities and ensure that all people with T1D have access to the best possible care. While significant progress has been made in the management of T1D, the journey towards an ideal, fully integrated and personalized treatment approach continues. The future holds the promise of more sophisticated management strategies, but it will require concerted efforts in research, technology development, and health policy.

## 8. Conclusions

The exploration of T1D and its associated complications in this review highlights the pivotal role of technological innovations, particularly in the form of Android applications and personalized hybrid closed-loop system (HCLS) algorithms. These advances are at the forefront of a revolution in diabetes management, offering new ways to mitigate the long-term complications of T1D. Central to this evolution is the integration of personalized algorithms into diabetes management. These are often only possible in open-source HCLS research frameworks today, but in the future will mark a significant leap toward individualized care, enabling more precise insulin dosing and reducing the risks associated with hyperglycemia and hypoglycemia. The potential of these applications to increase patient autonomy and engagement in their own care cannot be overstated. In addition, the development of personalized HCLS algorithms represents a major advance in the prevention of diabetes complications. By considering factors such as a patient’s activity level, dietary habits, and even hormonal fluctuations, these systems will be able to fine-tune insulin delivery to minimize the occurrence of glucose extremes (Figure 5). This level of personalization is also critical to reducing the incidence of micro- and macrovascular complications associated with T1D, ultimately improving patient outcomes and quality of life. In addition, the integration of these advanced technologies into diabetes management aligns with the broader shift toward precision medicine. By harnessing the power of big data analytics and machine learning, these systems can continuously evolve to adapt to changing patient needs and incorporate new research findings. This dynamic approach is key to staying ahead of disease progression and mitigating its complications.

In conclusion, the integration of personalized HCLS algorithms into the management of T1D is a game changer. It represents a new era of diabetes care that is more responsive, adaptive, and patient-centered. As these technologies continue to evolve, their role in the prevention and management of T1D complications will undoubtedly grow, opening the way for more effective and personalized healthcare solutions.

## Data Availability

No new data were created or analyzed in this study. Data sharing is not applicable to this article.
